# Mechanosensory encoding dysfunction emerges from cancer-chemotherapy interaction

**DOI:** 10.3389/fmolb.2022.1017427

**Published:** 2022-11-24

**Authors:** Stephen N. Housley, Paul Nardelli, Travis M. Rotterman, J’Ana Reed, Timothy C. Cope

**Affiliations:** ^1^ School of Biological Sciences, Georgia Institute of Technology, Atlanta, GA, United States; ^2^ Integrated Cancer Research Center, Georgia Institute of Technology, Atlanta, GA, United States; ^3^ W. H. Coulter Department of Biomedical Engineering, Emory University and Georgia Institute of Technology, Georgia Institute of Technology, Atlanta, GA, United States

**Keywords:** neurotoxicity, chemotherapy, sensory encoding, cutaneous, sensorimotor abnormalities, cancer treatment, proprioception

## Abstract

Persistent sensory, motor and cognitive disabilities comprise chemotherapy-induced neural disorders (CIND) that limit quality of life with little therapeutic relief for cancer survivors. Our recent preclinical study provides new insight into a condition impacting the severity of chronic CIND. We find that sensorimotor disability observed following cancer treatment exceeds that attributable to chemotherapy alone. A possible explanation for intensified disability emerged from evidence that codependent effects of cancer and chemotherapy amplify defective firing in primary sensory neurons supplying one type of low threshold mechanosensory receptor (LTMR). Here we test whether cancer’s modification of chemotherapy-induced sensory defects generalizes across eight LTMR submodalities that collectively generate the signals of origin for proprioceptive and tactile perception and guidance of body movement. Preclinical study enabled controlled comparison of the independent contributions of chemotherapy and cancer to their clinically relevant combined effects. We compared data sampled from rats that were otherwise healthy or bearing colon cancer and treated, or not, with human-scaled, standard-of-care chemotherapy with oxaliplatin. Action potential firing patterns encoding naturalistic mechanical perturbations of skeletal muscle and skin were measured electrophysiologically *in vivo* from multiple types of LTMR neurons. All expressed aberrant encoding of dynamic and/or static features of mechanical stimuli in healthy rats treated with chemotherapy, and surprisingly also by some LTMRs in cancer-bearing rats that were not treated. By comparison, chemotherapy and cancer in combination worsened encoding aberrations, especially in slowly adapting LTMRs supplying both muscle and glabrous skin. Probabilistic modeling best predicted observed encoding defects when incorporating interaction effects of cancer and chemotherapy. We conclude that for multiple mechanosensory submodalities, the severity of encoding defects is modulated by a codependence of chemotherapy side effects and cancer’s systemic processes. We propose that the severity of CIND might be reduced by therapeutically targeting the mechanisms, yet to be determined, by which cancer magnifies chemotherapy’s neural side effects as an alternative to reducing chemotherapy and its life-saving benefits.

## 1 Introduction

The severity of chemotherapy-induced neural disorders (CINDs) exhibits wide variability. Sensory, motor, and cognitive disabilities comprising CIND span the full range of clinical impairment scales assessed either in individual patients over time or within patient populations receiving standard-of-care treatment for the same cancer (Cavaletti and Marmiroli, 2010; Alcindor and Beauger, 2011). Although the origins of variable severity are not known, covariation with various factors including genetic variants proves prognostic (Seretny et al., 2014; Chua and Kroetz, 2017; Park et al., 2017). These observations suggest that CIND severity is mutable and responsive to constitutive biological factors, which, once identified, might be manipulated therapeutically to reduce CIND. This potential approach to treatment gains interest given that clinical management remains broadly ineffective apart from reducing or suspending chemotherapy at the cost of diminishing its anticancer benefits (Loprinzi et al., 2020).

Recent pre-clinical study from our laboratory reveals that neural dysfunction induced by chemotherapy in healthy rats is intensified in rats bearing colon cancer (Housley et al., 2020a). Disability caused by cancer and chemotherapy in combination exceeds their independent effects on a behavioral task relying on somatosensory perception and guidance of limb placement. The mechanism underlying this newly identified amplification phenomenon is left to speculation, possibly resulting from converging effects of chemotherapy and cancer’s systemic processes on the same biological processes, e.g., inflammatory, or metabolic (Housley et al., 2020a). Whatever the mechanism, our findings demonstrate that non-neural cancer boosts the severity of sensorimotor dysfunction in chronic CIND.

Patients rank impaired sensorimotor function among the most burdensome effects that persist following cancer treatment (Seretny et al., 2014; Sisignano et al., 2014; Avan et al., 2015). Common signs and symptoms include altered tactile and proprioceptive perceptions, defective gait and balance, as well as compromised manual dexterity (Bennett et al., 2012; Seretny et al., 2014; Sisignano et al., 2014). These disorders correspond with neuropathy exhibited by a class of primary somatosensory neurons that supply low threshold mechanoreceptors (LTMRs) and fire trains of action potentials encoding mechanical perturbations of skin and muscle. Multiple types of LTMRs are specialized to encode specific features, i.e., submodalities of tissue responses to diverse forms of mechanical perturbation. Signals generated by the population of responding LTMR neurons provide the central nervous system with composite mechanosensory information essential for normal tactile and proprioceptive experience and motor responses. Commonly used chemotherapy agents, e.g., taxanes and platinum-based compound, disrupt LTMR signaling. Disruption may occur when cancer treatment results in structural neuropathy, e.g., degeneration of LTMR distal axons (Fukuda et al., 2017). In addition, preclinical study demonstrates that cancer treatment induces a functional neuropathy in multiple types of LTMRs, wherein neuronal firing fails to encode biomechanical features of body touch and movement as it does normally (Housley et al., 2021). For one type, Ia muscle spindle neurons, we find that the severity of encoding defects emerges from codependent effects of cancer and chemotherapy (Housley et al., 2020a). Whether the severity of encoding defects is similarly amplified in any other LTMR type has not been examined.

In the present study, we tested the hypothesis that cancer’s exacerbation of encoding defects induced by chemotherapy propagates broadly across diverse mechanical submodalities served by LTMR neurons supplying muscle and glabrous skin. Firing responses encoding naturalistic mechanical perturbations were recorded electrophysiologically *in vivo* from multiple types of muscle and cutaneous LTMRs. Data were sampled and compared across four groups of rats: wild type rats vs. mutant rats bearing colorectal cancer, each receiving or not receiving a human-scaled course of chemotherapy with oxaliplatin, one of the platinum-based compounds commonly used in chemotherapy for wide rangingcancers (Galanski et al., 2005; Johnstone et al., 2014). Results revealed that codependent interaction of cancer and chemotherapy magnified defective mechanosensory encoding in all types of muscle and skin LTMR neurons, predominantly slowly adapting ones. The spread of codependent effects across diverse LTMR submodalities supports our hypothesis and suggests that targeting treatment to the systemic effects of cancer might achieve wide-sweeping reduction of CIND without suspending chemotherapy.

## 2 Materials and methods

### 2.1 Animals and experimental groups

All procedures and experiments were approved by the Georgia Institute of Technology Institutional Animal Care and Use Committee. Adult (250–350 g) female and male Fisher 344 (F344) (*Apc* ^*WT*^) rats and rats carrying a germline mutation *Apc gene* mutation (*Apc* ^*Pirc/+*^) (Amos-Landgraf et al., 2007) were studied after fully developed cancer is present at 4 months (Irving et al., 2014). Human-scaled dose of oxaliplatin treatment was initiated only after fully developed cancer is present (Housley et al., 2020a). All animals were housed in clean cages and provided food and water *ad libitum* in a temperature- and light-controlled environment. *Apc* ^*WT*^ + control, *Apc* ^*WT*^ + OX, *Apc* ^*Pirc/+*^+control, and *Apc* ^*Pirc/+*^+OX experimental groups (Tables 1, 2) herein referred to as: control, OX, cancer, and cOIN respectively. Due to differences in experimental preparations and requirements for different stimuli, different animals were utilized to study muscle vs. cutaneous LTMR neurons.

**TABLE 1:** Distribution of data. Breakdown of neuronal classes in top row and experimental group left most column.

225 muscle sensory neurons from 20 rats	Ia (*n*=60)	Unclassified (*n*=57)	II (*n*=57)	Ib (*n*=51)
Control (ApcWT, *n*=6)	11	19	17	14
OX (ApcWT+OX, *n*=3)	19	14	9	5
Cancer (ApcPirc/+, *n*=4)	20	19	17	18
cOIN (ApcPirc/++OX, *n*=7)	10	5	14	14

### 2.2 *In vivo* procedures

All treatments and *in vivo* procedures have been previously described (Haftel et al., 2004; Bullinger et al., 2011a; Nardelli et al., 2016; Nardelli et al., 2017; Vincent et al., 2017; Housley et al., 2020a). Briefly, 7 weeks after achieving clinically relevant chemotherapy doses, rats were deeply anesthetized initially by inhalation of isoflurane (5% in 100% O_2_), and for the remainder of the experiment *via* a tracheal cannula (1.5%–2.5% in 100% O_2_). Vital signs were continuously monitored including, core temperature (36–38°C), PCO_2_ (3%–5%), respiratory rate (40–60 breaths/min), pulse rate (300–450 bpm) and SPO_2_ (>90%). Lumbar dorsal roots together with muscles and nerves in the left hindlimb were surgically exposed and prepared for stimulation and recording as previously described (Vincent et al., 2017; Housley et al., 2020a). All other left hindlimb nerves were crushed to reduce extraneous neuronal activity. Individual axons in dorsal rootlets were penetrated by glass micropipettes and were selected for continuous intracellular study when electrical stimulation of triceps surae nerves produced orthodromic responses.

Subclasses of muscle LTMR neurons were distinguished as described in our earlier reports, e.g., (Vincent et al., 2017). Briefly, muscle neurons that fired during the rising phase of isometric twitch force were designated group Ib, while those that paused were classified as one of three types of muscle spindle neurons, including type Ia, type I unclassified (I_un_), or type II. Type II neurons were identified by a failure to fire with each cycle of muscle vibration (100 Hz frequency, 80 µm amplitude, and 1 s duration) and by little to no history-dependent firing when stretched by successive triplets of triangular stretches (3 mm, 4 mm/s). Both Ia and I_un_ neurons fired with 1-to-1 fidelity during vibration and exhibited significant history dependence. When presented with ramp-hold-release stretches (3 mm at 20 mm/s, 1 s hold), Ia neurons responded with an initial burst of high-frequency firing (>100 pps) at the onset of muscle stretch. Muscle LTMR neurons lacking an initial burst but exhibiting all other firing characteristics of type Ia neurons were classified as type I_un_. Spike trains generated by all muscle LTMR neurons were measured for several primary and derived parameters reported in Results.

In studies of cutaneous LTMR neurons, the surgical procedure outlined above was used, deviating only in that the post-tibial nerve was isolated and placed in-continuity within a bipolar stimulating cuff electrode; all other nerves in the left hindlimb were crushed. Calibrated von Frey filaments were touched to the glabrous skin on the bottom of the left foot to identify the force threshold and receptive field of LTMR cutaneous neurons. A servomotor operating in force-servo mode was used to produce skin displacement through a wooden dowel (2 mm diameter) applied to the most sensitive region of each recorded neuron’s receptive field. Two forms of skin displacement were studied: a ramp-hold-release (2 mm, 20 mm/s, 10 s hold) and vibration (100 Hz frequency, 80 µm amplitude, and 1 s duration). These mechanical perturbations evoked spike trains characterized by a variety of parameters, some of which were used to classify subtypes of cutaneous LTMR neurons. Slowly adapting neurons, including type I (SAI: Merkel corpuscles; Figure 4C) responded to constant skin displacement with non-uniform firing quantified by a high coefficient of variance (CoV), while type II neurons (SAII: Ruffini endings; Figure 4D) fired regularly (low CoV) throughout the hold phase. Rapidly adapting neurons, including ones supplying Meissner corpuscles (RA; Figure 4A) and Pacinian corpuscles (RA2 PC; Figure 4B) responded with brief firing at the onset of the stimuli lasting no more than 2 s and respond 1-to-1 to vibration. Meissner neurons were differentially identified by an additional short burst of firing during the release phase of the stimuli. Firing parameters measured from individual neurons are reported in Results. It was also possible to construct population codes compiled for each of the four experimental groups (Figure 7) by temporally aligning spike trains of multiple individual neurons which were recorded separately but evoked to fire by identical patterns of skin displacement. Similar study was applied to muscle LTMR neurons in our earlier report (Housley et al., 2021).

### 2.3 Statistical analysis

Principal Components (PC) provided unsupervised dimensionality reduction for the multiple features of neuronal signaling in an attempt to identify dominant patterns of covariation across neurons and treatments (Cunningham and Byron, 2014). PC analysis and visualization was performed with the *factoextra* (Kassambara and Mundt, 2016) and *FactoMineR* (Husson et al., 2010) in the R environment (4.0.3) (Team, 2015).

Linear discriminant analysis (LDA) provided supervised dimensionality reduction for the multiple features of neuronal signaling in attempt to find a linear combination of features that separated and characterized independent and combinatorial treatment effects (Supplementary Figure S2). Data were first de-meaned, normalized to unit standard deviation, and tested by Bayesian one-way ANOVA (*stan_glm*) (Gabry and Goodrich, 2018). The derived covariance matrix was then normalized by within-group, pooled covariance. The eigenvectors of that modified covariance matrix defined three canonical variables that characterized and separated the four treatment groups. LDA and 10-fold cross validation of model performance (repeated holdout method) was performed with the *MASS* (7.3–51.1) library in the R environment (4.0.3).

All other statistical techniques for evaluating neuronal encoding have been described in previously published reports from this laboratory (Horstman et al., 2019; Housley et al., 2020a). Briefly, Bayesian parameter estimation was used to derive the entire joint posterior distribution of all parameters simultaneously for statistical comparison. Highest (posterior) density interval (HDI) was used to make unbiased inferences by directly comparing the posterior probability distributions (95%) between two (or more) contrasts of interests e.g., mean-comparisons testing (Horstman et al., 2019; Housley et al., 2020a). All models were developed with the *rstanarm* package (2.21.1) (Gabry and Goodrich, 2018) in R (4.0.3) (Team, 2015). Models were validated by computing out-of-sample predictive accuracy using Pareto-smoothed importance sampling [PSIS (Vehtari et al., 2017)] to perform leave-one-out cross validation as previously described (Housley et al., 2020a; Housley et al., 2020b). Summary statistics of observed data are reported as mean ± SE.

## 3 Results

### 3.1 Codependent effects of cancer and chemotherapy exacerbate sensory encoding impairment in all types of muscle low threshold mechanosensory receptor neurons

We recently identified significant functional encoding deficiencies across a range of LTMR neurons in response to chronic cancer treatment (Housley et al., 2021). However, the extent to which defects depend on the joint or independent effects of cancer and/or its treatment (Housley et al., 2020a) across the diverse set of LTMRs was unknown for all types except type Ia muscle spindle neurons. For Ia’s, we discovered robust evidence for codependent effects of cancer and chemotherapy distributed across genetic, protein, sensory encoding, and behavior (Housley et al., 2020a). In order to test this possibility in other classes of muscle LTMR neurons, we performed *in vivo* electrophysiological studies on type I unclassified and type II muscle spindle neurons and on type Ib tendon organ neurons that variously encode muscle dynamics, i.e., unique static and time-varying parameters of muscle biomechanics measured as muscle force, position, velocity, and stiffness (Vincent et al., 2017).

**FIGURE 1:**
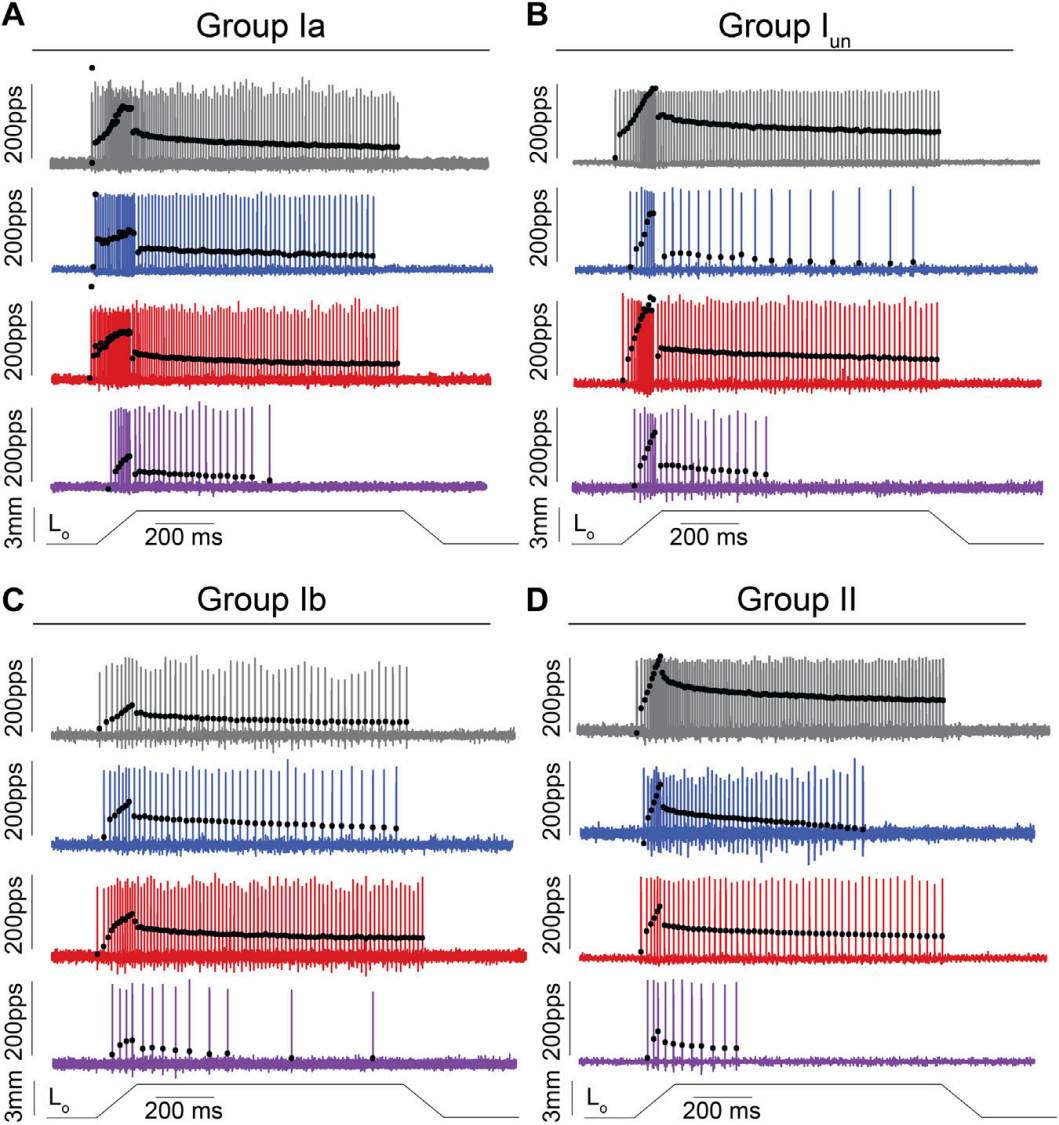
Impaired Muscle Mechanosensory Neuron Population Code After Cancer Treatment. **(A–D)** Representative cases of spiking activity in control (grey), OX (blue), cancer (red), and cOIN (purple) as a measure of sensory encoding in spindle group Ia **(A)**,unclassified spindle **(B)**, group Ib **(C)**, group II **(D)**. Blackcircles plot instantaneous firing rates (IFR) measures as pulses per second (pps) of corresponding spike (action potential: vertical lines) intervals. Dashed vertical line marks onset of muscle stretch [3 mm from resting length (Lo)] shown in bottom trace divided into dynamic and static phases by dark grey (150 ms duration after stretch command onset) and light grey (1 s duration after the dynamic phase) bars.

**FIGURE 2:**
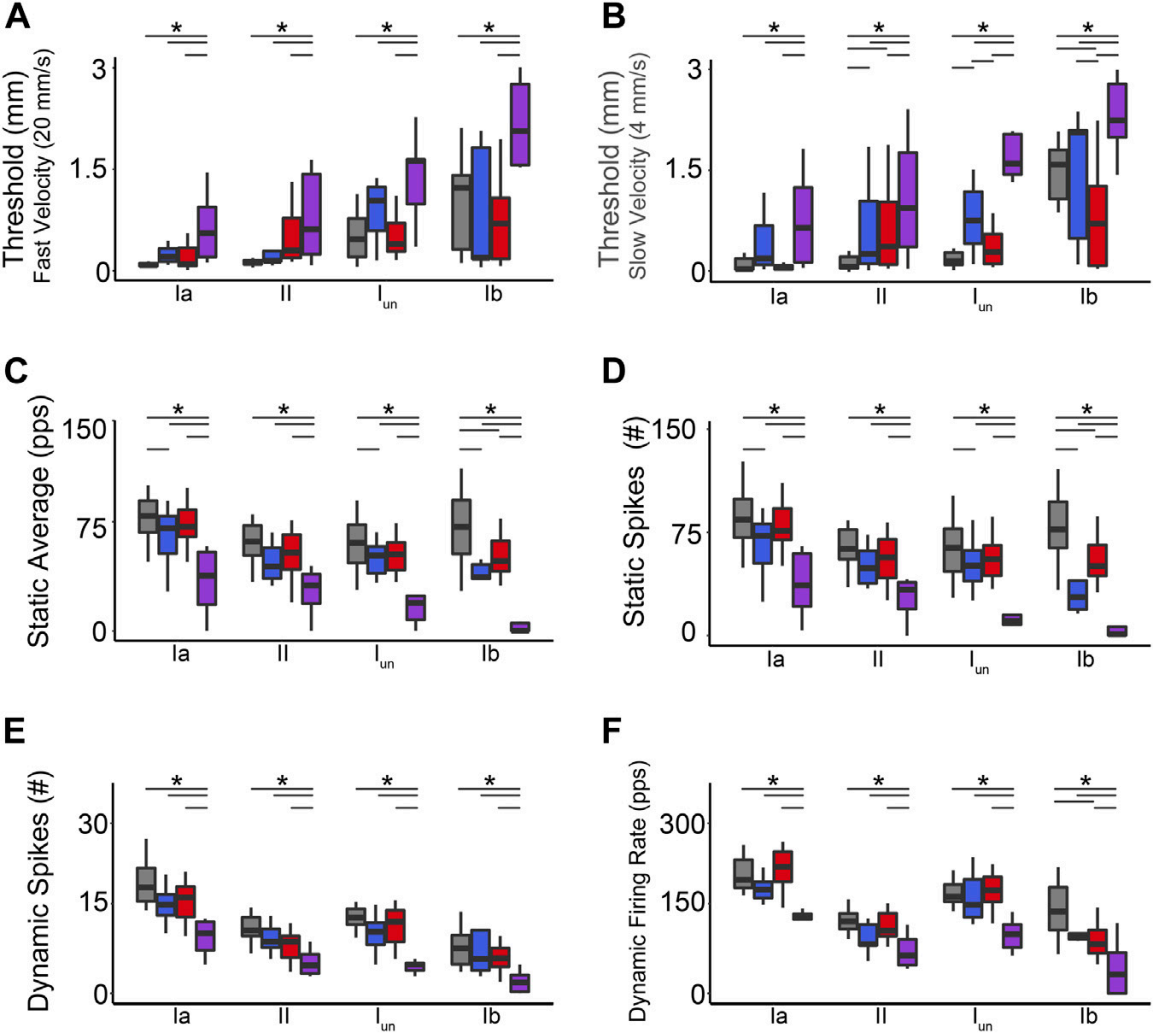
Co-Impairment of Key Muscle Mechanosensory Features. Quantification of clusters of encoding parameters: detection threshold: during fast **(A)** and slow **(B)** stimuli, static average **(C)**, number of spikes during static **(D)** and dynamic stimuli **(E)**, and peak dynamic firing rate **(F)**, averaged from four trials, from each neuron in each of the neuronal classes. * indicates statistically significant differences as empirically derived from hierarchical Bayesian model (stan_glm): 95% highest density intervals do not overlap.

We recorded the spiking activity evoked by naturalistic muscle stretch from a total of 225 LTMR neurons sampled from four experimental groups (Table 1). Data from type Ia neurons are reproduced from our previous study (Figures 1, 2) (Housley et al., 2020a) to aid comparison with the other LTMR types. From spiking responses averaged over four stretch trials, we collected 31 measured and derived parameters (Supplementary Figure S1; Supplementary Table S1). We first asked whether cancer or OX treatment alone altered neuronal signaling. In agreement with analyses of type Ia neurons (Housley et al., 2020a), we found a remarkable degree of similarity in neuronal signaling between cancer (Figure 1 red traces) and control (Figure 1 grey traces). As was true for group Ia neurons, OX treatment induced mild signaling in the other types. Deficits were primarily restricted to sustained firing (Figure 1 blue traces), validating our previous findings in a different strain of healthy rats treated with OX alone (Vincent et al., 2016). Hierarchal Bayesian modeling was then used to quantify inferences drawn from raw data (Figure 2). Across the majority of parameters and neuron types, we found that while cancer and OX do induce signaling alterations, their effects alone were not sufficient to explain their combined effects observed in cOIN. Interestingly, the relationships among encoding parameter values between LTMR neuron types was conserved across all experimental groups. This suggests that either a global co-regulatory process may be present that governs the balance of information content flowing to the central nervous system from multiple neuron types and/or the different types are similarly affected by OX and cancer. By contrast, we found that Ib neurons in the cancer group exhibited lower threshold (more responsiveness) to slower velocity movements (Figure 2B) as compared to control or OX groups alone, which is the sole exception to the well conserved balance among neurons and their effects. Collectively, these findings suggest that the effects of cancer or OX alone are insufficient to explain the firing abnormalities observed in cOIN (Figure 1 purple traces), thereby supporting the hypothesis that codependent interaction significantly exacerbates signaling deficits across all muscle LTMR neurons.

### 3.2 Comparable effects across disparate muscle low threshold mechanosensory receptor neurons

**FIGURE 3:**
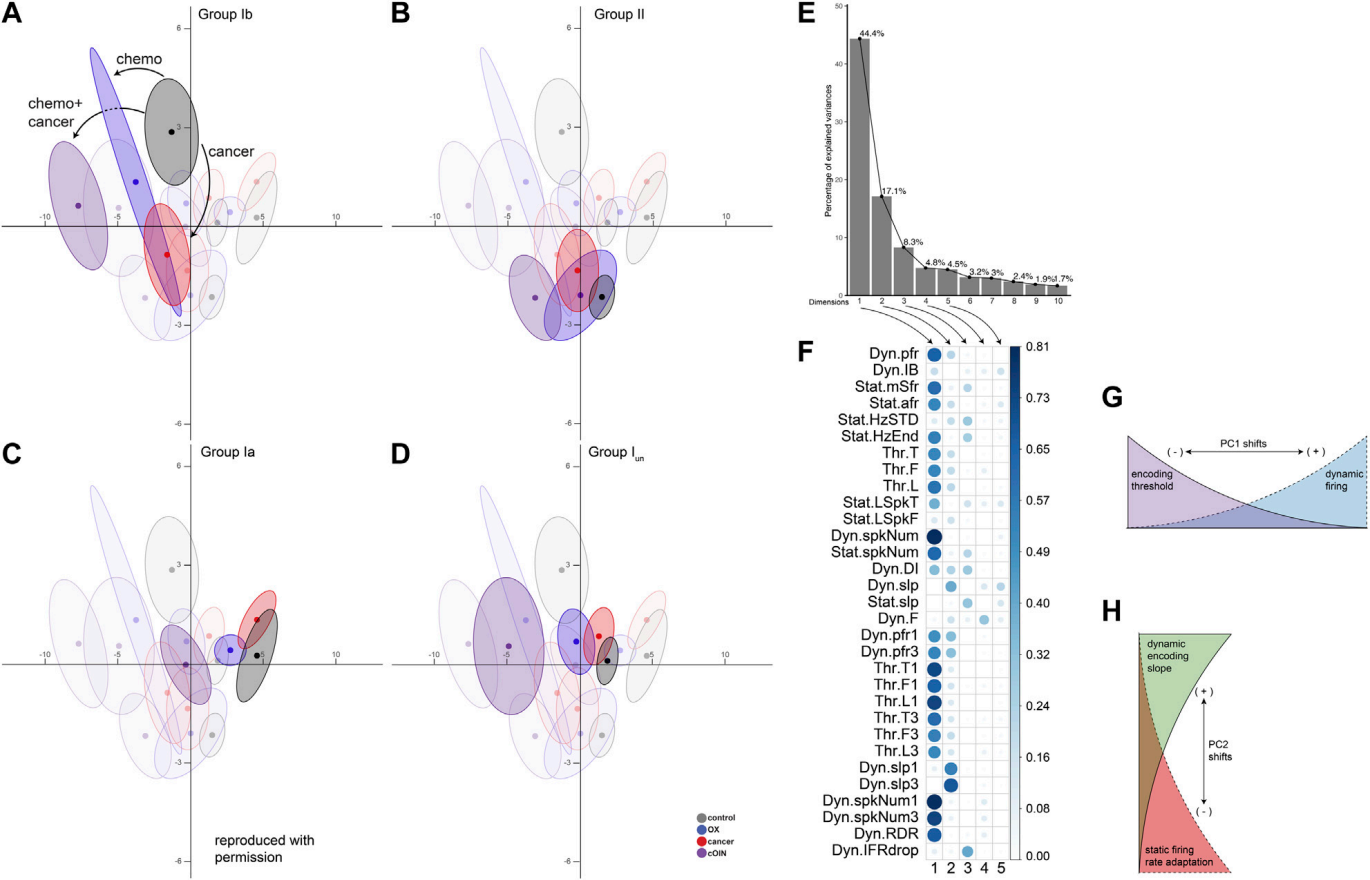
Latent encoding space reveals unique effects of cancer, chemotherapy and their combination across divergent classes of propriosensors. Principal component (PC) analysis applied to all (*n* = 31) encoding parameters measured in response to natural stimuli for each neuron class emphasizing: spindle group Ib **(A)**, group II **(B)**, group Ia **(C)**, unclassified spindle **(D)** with greater opacity. Neuron class means (average of PC1-2 coordinates across all neurons in a given class and treatment group: dark colored circles) are visualized in the new latent encoding space created by PC1–2. Color-coded least-squares elliptical fitting (95% confidence) was computed to emphasize differences between neuron classes and experimental groups. Labelled arrows in **(A)** indicate the magnitude and direction of cancer, chemotherapy, or their combined effects. **(E)** Scree plot indicating percentage of explained variance by each PC (grey bar, eigenvalue in %). **(F)** Corrplot indicates the contribution of variables on the factor map for PC1-5. Larger values indicate the components contribute a larger relative portion, indicating components are of greater importance. **(G,H)** Summarize the effects of shifting along principle axes in latent encoding space.

We then characterized defects in neuronal signaling across all muscle cell types in a common stimulus encoding subspace. We exploited principal component (PC) analysis for its potential to uncover latent patterns in the 31 measured and derived encoding parameters to provide a parsimonious description of statistical features of interest, e.g., a common framework for comparing the effects of cancer, OX, and their combination (cOIN) on different types of muscle LTMRs (Cunningham and Byron, 2014). PC analysis identified a low-dimensional encoding space (PC1-2) in which the majority of observed treatment effects (61.5%) are conserved across all neuron types (Figures 3A–D) and treatment groups (Figures 3A–D color coded ellipses). Cell type means, 95% confidence ellipses, and arrows are drawn to indicate the magnitude and direction of treatment effects for each neuron type. We highlight (through altering opacity) each neuron class in the global encoding space (Figures 3A–D) to clarify interpretation.

Scree plot (Figure 3E) depicts the independent variance accounted for by each of the top ten PCs. Figure 3F indicates the parameters that influence PC1-5, where larger values contributed a larger portion to their respective PC scores. In the OX group, neuron encoding space shifts were principally along PC1 for the super-family of muscle spindle neurons (types Ia, Iun, and II), although not significant for type II neurons. Leftward shifts corresponded with decreased dynamic firing and increased detection threshold, i.e., lower sensitivity to muscle stretch (cf Figure 3G). Cancer effects alone were more complex, comprising shifts along both PC1 and PC2 axes. Both Ia and Ib neurons responded to cancer in PC2. However, the PC2 shift for Ib neurons was significantly larger and in the opposite direction compared to Ia neurons. Specifically, Ibs responded to cancer with increased static firing adaptation and decreased representation of dynamic encoding, i.e., decreased excitability in opposition to encoding changes observed for Ia neurons (cf Figure 3H).

This global approach utilizing all encoding parameters identified three key observations. First, our analysis revealed effects of OX and cancer distributed across latent encoding space that would otherwise be covert to single parameter analysis. Second, the spindle super-family (Ia, I_un_, and II) maintained a relative orientation in the latent encoding space throughout the independent effects of cancer and OX and their joint effects in cOIN. Finally, these findings provide additional empirical and analytic corroboration that independent effects of cancer and OX are insufficient to explain the clinically relevant effects of cancer treatment.

### 3.3 Generative modeling provides definitive evidence of co-dependent effects

To quantify inferences drawn from raw data, we took an unbiased statistical approach by subjecting all signaling parameters from all neurons, types, and experimental groups to a LD analysis. This reduced complex feature space into canonical variables giving us a high-level understanding of where interaction emerges, without biased parameter selection *a priori*. Our analysis yielded three canonical variables that achieved overall 98.7% classification accuracy (Supplementary Figure S1). We then visualized neuronal signaling in the new 3D composite space created by LD1-3 (Supplementary Figure S1) revealing a first dimension (LD1) that accounted for a large fraction of the variance for each class of neurons, ranging from 64% to 66% (Figures 2D–F). Coincidentally, LD1 also represented the non-linear interaction between cancer and OX, that can be visualized by the 3D separation of the purple spheroid in LD1 [as was the case for group Ia (Housley et al., 2020a)].

**FIGURE 4:**
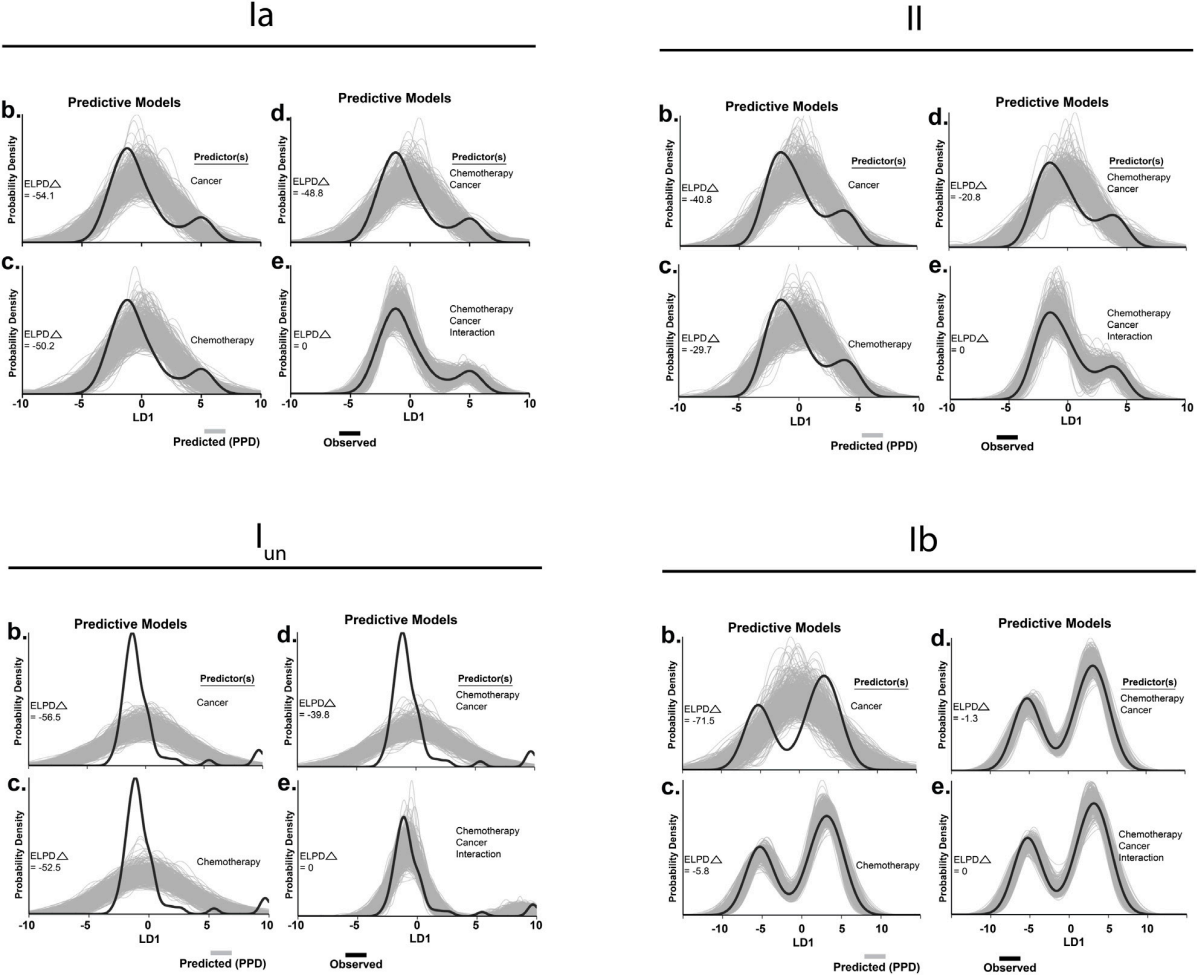
Hierarchical Bayesian modeling. Tests for significant group differences in LD1 scores reconfigured to operate in a predictive fashion in spindle group Ia **(A)**, group II **(B)**, unclassified spindle **(C)**, group Ib **(D)**. Predictors included in each model are listed to the right of each subplot (1–4). Generative models in utilizing one (1 and 2) or both independent 3) predictor(s) for posterior prediction. The generative model in 4) utilizes both independent predictors and an interaction term for posterior prediction. Grey lines in represent 500 novel (generative) samples drawn from the posterior distributions. Black lines illustrate experimentally observed mean LD1 score. Predictive accuracy was measured by calculating expected log predictive density (ELPD) for each model and benchmarked off of the highest performing model. Delta ELPD (*Δ*) indicates difference from optimal model. Negative models represent worse predictive performance.

To test the statistical significance of the co-dependent effects of cancer and OX, we conducted Bayesian model comparison with full factorial and all restricted models using leave-one-out cross validation. We quantified and validated each model’s predictive performance by computing the expected log predictive densities (ELPD; measure of a model’s out-of-sample predictive accuracy in Figures 4A–D). While we found decisive evidence in favor of models including a cancer–OX interaction predictor (fourth panel in each of Figures 4A–D), the interaction effect parameter proved more impactful for the superfamily of spindles as compared to group Ibs (Figure 4D). From these data and analytical methods, we conclude that co-dependent effects (interaction) of cancer and OX were required to accurately reproduce encoding deficits in cOIN.

### 3.4 Codependent effects of cancer and chemotherapy exacerbate sensory encoding impairment in all types of glabrous cutaneous low threshold mechanosensory receptor neurons

**FIGURE 5:**
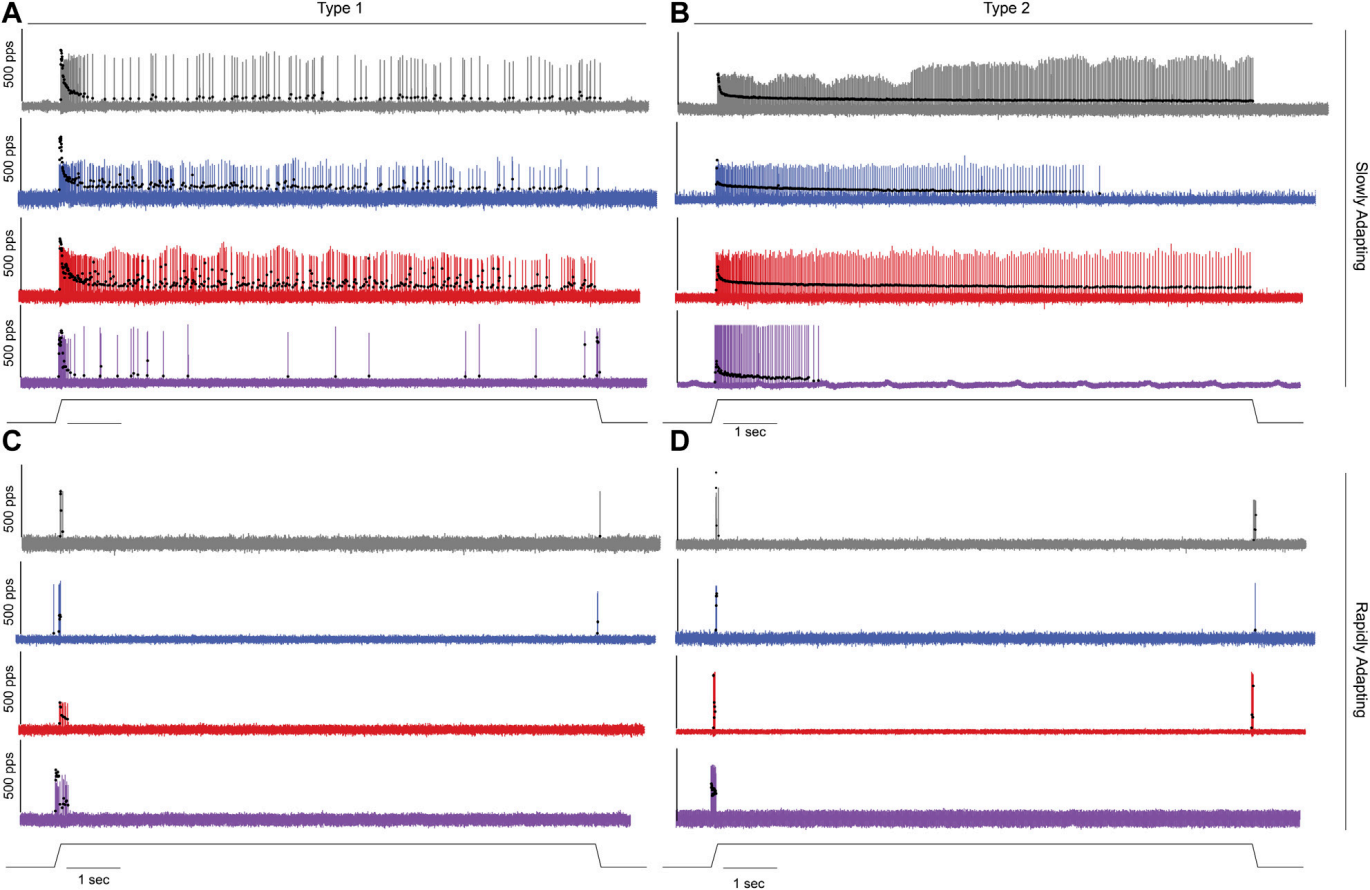
Impaired Cutaneous Mechanosensory Neuron Code After Cancer Treatment. Representative cases of spiking activity in control (grey), OX (blue), cancer (red), and cOIN (purple) as a measure of sensory encoding in slowly adapting type I SA1: Merkel corpuscles; **(A)** and type II SA2: Ruffini endings; **(B)** and rapidly adapting Meissner corpuscles RA1; **(C)** and Pacinian corpuscles RA2; **(D)**. Black circles plot instantaneous firing rates (pps) of corresponding spike (action potential) intervals. Solid line below voltage recordings indicates the dynamic and static components of the natural stimulation paradigm, i.e. displacement of the plantar skin *in vivo* utilized to study the cutaneous neurons (1 mm from resting length: Lo).

We recently identified significant encoding deficiencies across four classes of cutaneous LTMR neurons in response to chronic cancer treatment (Housley et al., 2021). However, the extent to which defects depend on the joint or independent effects of cancer and/or its treatment (Housley et al., 2020a) was unknown. To fill this gap in knowledge, we recorded from LTMR neurons supplying slowly adapting (SA) Merkel corpuscles (SA1; Figure 5A) and Ruffini endings (SA2; Figure 5B) and rapidly adapting (RA) Meissner corpuscles (RA1; Figure 5C), and Pacinian corpuscles (RA2; Figure 5D) by applying pressure to the plantar skin of the hind-foot *in vivo* (Methods). While encoding by cutaneous SA1, SA2, RA1, and RA2 neurons was largely distinguishable based on their signaling characteristics (Leem et al., 1993) (Figure 5, see Methods: Table 2), we observed neuron-type by treatment specific disruption in their canonical encoding phenotypes as compared to control (Figure 5). The following sections highlight specific signaling characteristics and the unique changes induced by cancer and/or chemotherapy.

**TABLE 2:** Distribution of data. Breakdown of neuronal classes in top row and experimental group left most column.

173 cutaneous sensory neurons from 28 rats	SA1 (*n* = 50)	SA2 (*n* = 19)	RA1 (*n* = 76)	RA2 (*n* = 28)
Control (Apc^WT^, *n* = 13)	18	7	38	12
OX (Apc^WT^ + OX, *n* = 4)	14	6	21	7
Cancer (Apc^Pirc/+^, *n* = 3)	11	3	10	3
cOIN (Apc^Pirc/+^+OX, *n* = 8)	7	3	7	6

#### 3.4.1 Threshold

**FIGURE 6:**
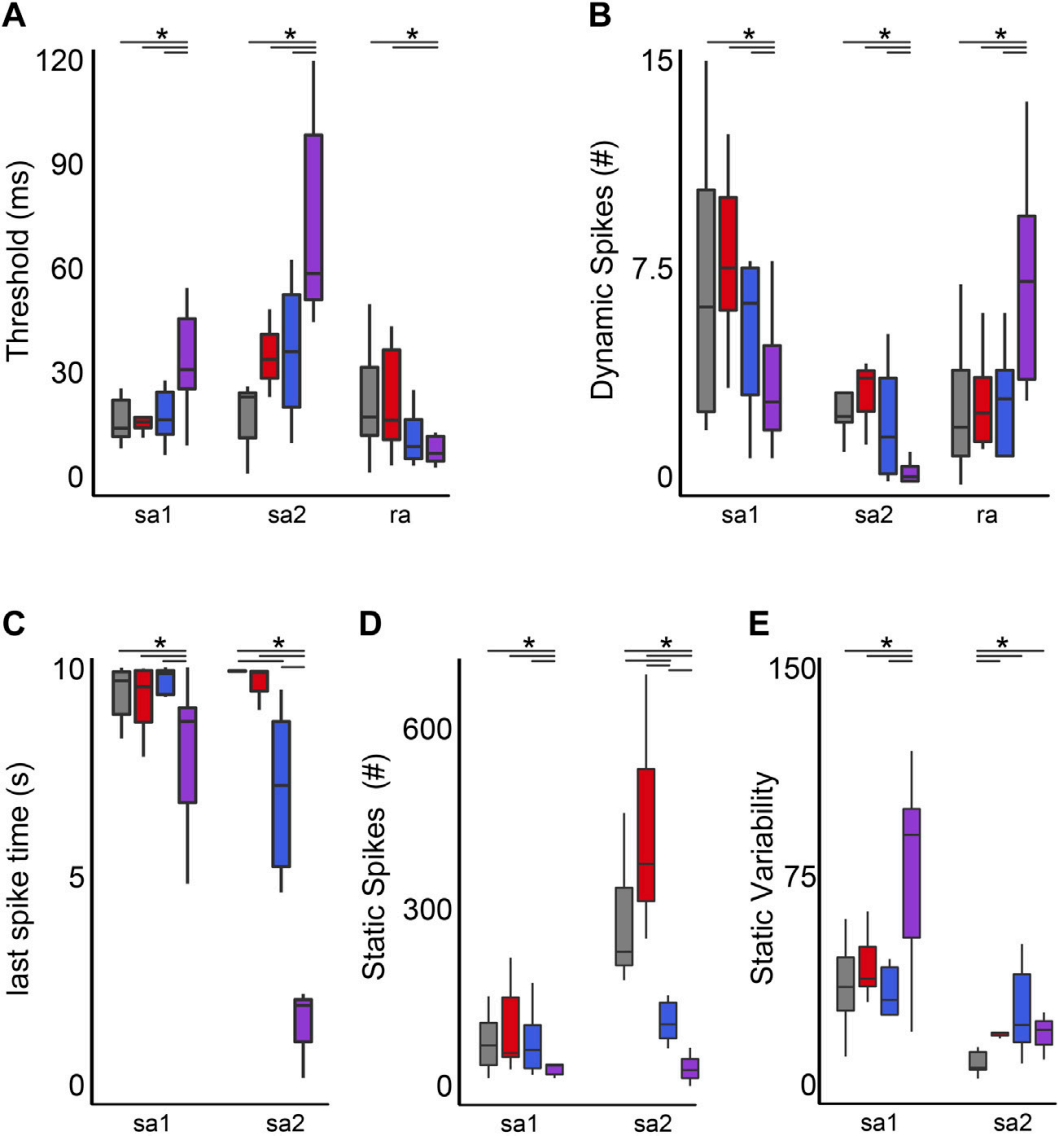
Co-Impairment of Key Cutaneous Mechanosensory Features. Quantification of clusters of encoding parameters: detection threshold **(A)**, dynamic spike encoding **(B)** last spike time **(C)**, number of spikes during static **(D)** and spike variability during static stimulus presentation **(E)** averaged from four trials, from each neuron in each of the neuronal classes. * indicates statistically significant differences as empirically derived from hierarchical Bayesian model (stan_glm): 95% highest density intervals do not overlap.

Stimulus detection (threshold) in RA neurons did not differ between cancer and control but was reduced by OX regardless of the presence of cancer (Figure 6A). While stimulus detection in SAII neurons was increased by the independent effects of cancer or OX, defects were significantly less than impairments observed in cOIN (Figure 6A). Changes in SAI neuron threshold were uniquely observed in cOIN as no deficiencies were detected from the independent effects of OX or cancer.

#### 3.4.2 Dynamic firing

Reduction in the group-level dynamic firing rates of SA1 and SA2 neurons emerged only in cOIN (Figure 6B). It should be noted that a minority (*n* = 2/6) of SA2 neurons recorded from OX animals mirrored the effects observed in the more consistently impaired cOIN group. Surprisingly, we detected significant increase in the dynamic firing capacity of both RA neuron types (Figure 6B) in cOIN, whereas no changes were detected in OX or cancer.

#### 3.4.3 Static firing duration, rate, and variability

The capacity to sustain firing in response to constant skin displacement (Figure 5) in SA1 and SA2 neurons was dramatically impaired in cOIN (Figure 6C). We did not observe changes in the duration of firing of SA1 and SA2 neurons recorded from animals with cancer, nor did we detect changes in SA1 firing duration in OX. OX treatment significantly reduced SA2 firing duration yet was substantially longer than cOIN (Figure 6C). Investigation of the static firing rates across SA neurons corroborated the previous findings (Figure 6D) with one exception; cancer significantly increased static firing rates as compared to control (Figure 6D). cOIN uniquely increased the static firing variability in SA1 neurons. The low static firing variability, a hallmark of SA2 neuron encoding, was equally increased in cancer, OX, and cOIN (Figure 6E). Note that increased firing probability for SA2 neurons did not reach levels exhibited by control SA1 neurons. For that reason, our designation of SA2 neurons after cancer treatment seemed justifiable, although we cannot completely rule out the possibility of misclassification.

### 3.5 Cutaneous population encoding

**FIGURE 7:**
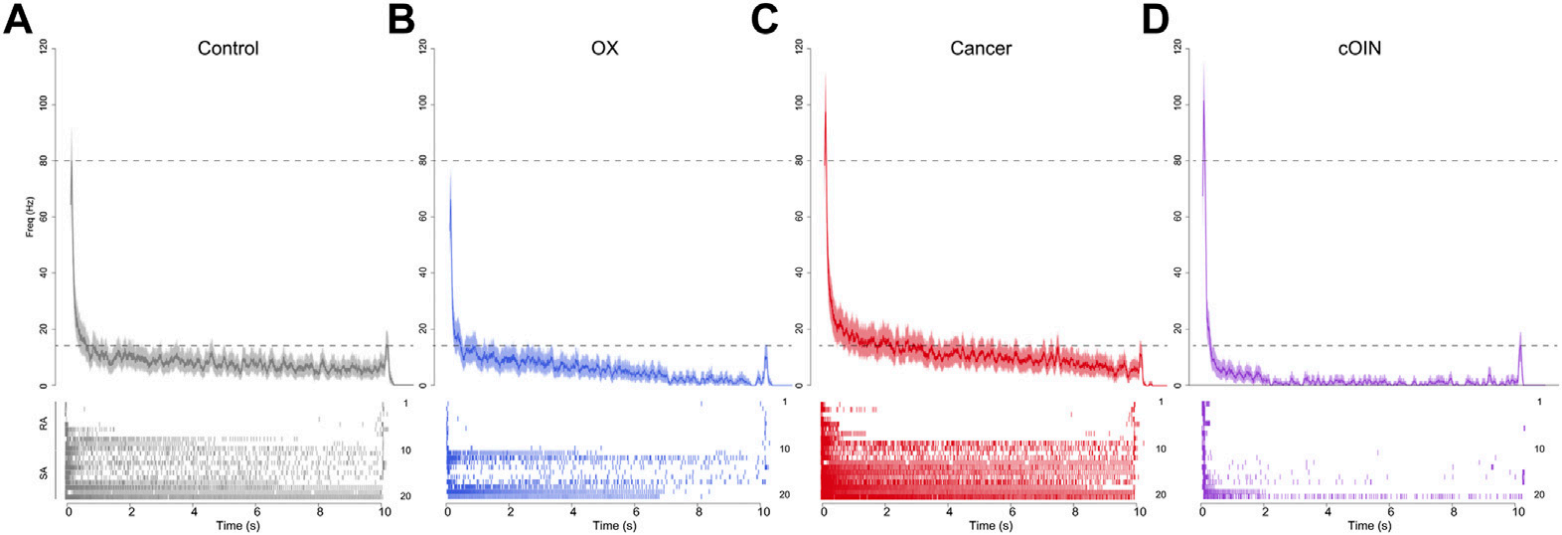
Chemotherapy for cancer impairs cutaneous population encoding. Population code of 20 neurons recorded from control (grey, **(A)**, OX (blue, **(B)**, cancer (red, **(C)**, and cOIN (purple, **(D)**. Raster plots bottom panels: **(A–D)** stack representative firing responses of 20 different afferents (1 afferent per row) aligned on identical ramp-hold-release displacement applied to each afferent’s glabrous cutaneous receptive field. Top traces show average firing rate (solid colored lines) and standard error (shaded region) computed per experimental group from the corresponding raster. Population codes for each group were constructed from the same distribution of afferent types: seven slowly adapting type I (SAI: Merkel corpuscles), 3 type II (SAII: Ruffini endings), seven rapidly adapting Meissner corpuscles (RA RA1) and three Pacinian corpuscles (RA2 PC).

Next, we simulated the more naturalistic condition wherein skin displacement was encoded by a neuronal population of multiple submodalities. For control, OX, cancer, and cOIN rats (Figure 7), we analyzed an ensemble of spike trains compiled from 20 randomly sampled neurons. The average firing rate profile compiled from a population of cutaneous neurons in cOIN rats displayed considerable differences from control, OX, and cancer in representing skin displacement (Figure 7). Prominent deficits in the population code included, exaggerated dynamic encoding (firing rate at the peak of changing skin displacement) (Figure 7 upper dotted horizontal line) and rapid and nearly complete accommodation after the first two seconds of sustained displacement. Surprisingly, the detection of stimulus offset, a canonical firing feature of RA neurons was intact (Figure 7 lower dotted horizontal line). This constellation of unique degeneracies represents a joint exacerbation of impairments exceeding a simple linear sum of changes observed independently in cancer and OX. Notably, elevated static firing rates were clearly observed in the cancer population code, as previously predicted (Figure 6D).

Collectively our findings demonstrated substantial and often heterogeneous alterations among the cutaneous sensory encoding modalities that provide plausible mechanistic explanations of the diverse sensory symptoms experience by patients, independent of degeneration.

## 4 Discussion

Our results show that platinum chemotherapy and colon cancer in combination exceeded their independent effects on neuronal encoding of mechanosensory information. This first corroboration [cf. Housley et al. (2020a)], establishes codependent intensification of neuronal encoding defects among the few known conditions impacting the severity of sensorimotor impairment in CIND. Here we uncovered evidence of codependent intensification for all LTMR neurons in both muscle and glabrous skin. We predict that widespread intensification of encoding defects at the very origin of mechanosensory signaling will necessarily impact the severity of tactile and proprioceptive disabilities induced by cancer treatment. Furthermore, we propose that managing cancer’s contribution to chemotherapy-induced neural disorders has the potential to diminish defective mechanosensory encoding and, in turn, significantly reduce chronic sensorimotor disability without withholding chemotherapy.

### 4.1 Severity of mechanosensory defects emerges from codependent effects of cancer and chemotherapy

Our objective was to determine whether the codependent effects of cancer and chemotherapy that intensify encoding defects extend over the full range of LTMR submodalities. Meeting this goal required comparison of encoding defects induced independently and jointly by chemotherapy and cancer. Preclinical study had the advantage of being unbounded by the clinical reality that patients without cancer rarely receive chemotherapy. In otherwise healthy rats, we identified independent effects of chemotherapy resembling those we reported earlier for a different rat strain (Bullinger et al., 2011b; Vincent et al., 2016). Among several individual and derived encoding parameters, the duration of static firing was most affected, being significantly reduced for all slowly adapting muscle and skin LTMRs. Analysis of latent space derived from multidimensional analysis of all parameters, available only for muscle LTMRs (see Methods), revealed shifts toward increased detection threshold and decreased dynamic firing, i.e,. diminished excitability for all four types of muscle LTRMs (Figure 3). In no case did the encoding defects induced by chemotherapy in any muscle or skin LTMR reach the levels observed following cancer treatment. In other words, the pervasive mechanosensory encoding defects and associated disabilities attending cancer treatment in cOIN rats were not caused by the independent side effects of chemotherapy alone.

The independent effects of untreated cancer on LTMR encoding were even smaller than the modest effects of chemotherapy alone. Measurements of single encoding parameters revealed almost no differences from untreated control rats. One exception was an anomalous increase in firing rate of slowly adapting cutaneous LTMRs. Also, multidimensional analysis uncovered changes in dynamic and static firing that were exaggerated in type Ib muscle LTMRs. Again, these changes fell short of those observed in cOIN. Nonetheless, the results also demonstrate that systemic processes of non-neural cancer can independently induce neural dysfunction. This observation aligns with demonstrations of cognitive disorders and associated brain abnormalities both clinically and preclinically induced by cancer in the absence of chemotherapy (Vardy et al., 2015; Kovalchuk et al., 2017).

From observations of independent effects, we infer the magnitude of neuronal dysfunction emerges from some codependent effect of cancer and chemotherapy that encompasses all LTRMs and perhaps other sensory modalities, e.g., temperature and pain. While these data, which corroborate our past report (Housley et al., 2020a), firmly established that codependent effects of chemotherapy and cancer influence neuronal function, the exact site(s) of this interaction remain unknown. It is important to recognize that neither process is stationary with respect to time, e.g., development of cancer, accumulation of chemotherapy, and subsequent suppression of cancer, suggesting that future studies should attempt to uncouple the temporal pattern by which these two systemic perturbations work together to influence pathophysiology of CIND. The magnitude and breadth of dysregulation we observed leads us to suspect that codependent interactions may be mediated by high-levels of biological control. Such candidates include DNA methylation and hydroxymethylation, histone modification, e.g., acetylation and deacetylation and non-coding RNA regulation, e.g., miRNAs, all of which comprise global regulatory processes known as epigenetic phenomena (Kim et al., 2009). While it is clear that perturbation of epigenetic control may lead to alterations in gene expression, cellular transformation, and ultimately cancer development (Lund and van Lohuizen, 2004; Esteller, 2008), it is less clear whether cancer itself can cause alterations to epigenetic control either directly or indirectly *via* systemic signaling pathways [attention is focused on indirect mechanisms since this model does not have metastatic disease which invades the nervous system (Amos-Landgraf et al., 2007; Irving et al., 2014)]. Identifying the existence of such reciprocal relationships may be crucial to determine where feedback loops intersect with the known influences of chemotherapy on epigenetic control in the nervous system of animals in preclinical study, (Briones and Woods, 2011; Wang et al., 2015). While the evidence is far from conclusive, we hypothesize that the administration of chemotherapy agents in the presence of systemic influence of cancer initiates a cascade of biological changes, with transient alterations in low levels of biological control, e.g., hyperexcitability: a positive acute effect observed throughout the nervous system and changes in inflammatory milieu that ultimately converge on high level epigenetic alterations that persist long after treatment cessation and disease free survival. These epigenetic changes would then lead to gene expression changes, altering metabolic activity and neuronal alterations that are responsible for driving CIND. Effects of epigenetic alterations may also help explain heterogeneity in patient populations (Lyon et al., 2014).

Alternatively, intermediate or lower levels of biologic control might be the center of codependent interactions between cancer and chemotherapy. Our global transcriptional analysis afforded some insight into this possibility pointing to changes in regulators of voltage-gated ion channels, e.g., oxidative, inflammatory, and metabolic pathways or voltage-gated ion channels themselves as likely culprits (Housley et al., 2020a). While previous studies suggest changes to systemic inflammatory milieu are transient (Wang et al., 2015), our data identify persist effects many weeks after treatment cessation. Moreover, the expression of persistent changes in low levels of biologic control simply rules in their potential role and does not rule out the possibility that they emerge from high-level epigenetic alterations that this or our prior (Housley et al., 2020a) work cannot currently disentangle.

### 4.2 Defective low threshold mechanosensory receptor encoding following cancer treatment predicts disability

Signals encoded by LTMRs are a major and direct source of mechanosensory information about kinematics and kinetics of body movement and posture, and about shape, size, weight, grip, and textures of objects (Shenton et al., 2004; Proske and Gandevia, 2012; Abraira and Ginty, 2013). From its origins in LTMRs, this information distributes widely throughout the CNS where it is processed to generate proprioceptive and tactile perceptions and to guide, correct, and predict movements appropriate for responding to or anticipating mechanical stimuli (Shenton et al., 2004; Proske and Gandevia, 2012; Bohic and Abraira, 2022). Vision and vestibular systems contribute partially overlapping information, but certainly not for body movements that occur outside the visual field or that minimally disturb gravitational loads (Shenton et al., 2004; Blanchard et al., 2013). Disruption of LTMR signaling predicts, therefore, disability with the sensorimotor behaviors described above. To a first approximation, disorders in CIND follow those predictions. Common signs and symptoms include paresthesias, reduced tactile senses, and diminished manual dexterity, as well as compromised gait, balance, and skilled movements (Wang et al., 2021; Wang et al., 2022). This litany of disability fits with the spread of dysfunction to multiple LTMR submodalities. We might expect some distinction in behavioral disorders in line with more severe encoding defects expressed by slowly adapting vs rapidly adapting LTMRs. Further speculation requires caution given inadequate understanding of the translation of mechanosensory signals into sensorimotor behaviors. Nonetheless, human disease states, e.g., large fiber neuropathy, and experimental deletions that selectively reduce or eliminate various LTMRs produce deficits in balance, gait, and skilled movements resembling those observed in CIND (Akay et al., 2014; Macefield, 2021).

Sensorimotor disability in CIND might arise from two types of peripheral neuropathy. Our preclinical studies identify encoding errors, whereby LTMRs generate action potentials and conduct them to the CNS, but aberrant firing misrepresents mechanical stimuli. The abnormally delayed and abbreviated firing we find, for example, would necessarily contribute to balance and gait impairments often suffered by patients following cancer treatment (Van Der Kooij and Peterka, 2011; Kneis et al., 2016; Schmitt et al., 2017). Alternatively, clinical diagnoses and studies regularly attribute sensorimotor disorders in CIND to degeneration of sensory terminal axons (Cavaletti and Marmiroli, 2010). In this condition, complete elimination of signaling by the affected fraction of LTMRs should also yield disabilities similar though distinguishable from those caused by encoding errors (Housley et al., 2021). However, axonal degeneration alone appears insufficient to fully explain disability. Clinical studies collectively describe inconsistent results in the relationship between patient-reported disability and estimates of physical degeneration taken from epidermal biopsy and peripheral nerve conduction studies (Velasco et al., 2014). In the extreme, patient-reported disability occurs in the absence of axon degeneration (Burakgazi et al., 2011). While sensory encoding errors have not yet been tested clinically, their occurrence is consistent with sensorimotor disorders we report for patients following cancer treatment (Wang et al., 2022). Patients exhibit errors in a force-matching task performed at the shoulder, which is unlikely to undergo dying-back neuropathy, because of its proximal location in the limb (Fukuda et al., 2017). In our preclinical model, and possibly also in patients, encoding defects occur independently from structural degeneration (Bullinger et al., 2011b; Housley et al., 2020a). We propose, therefore, that both structural and functional neuropathies contribute to CIND. Whether chemo-cancer codependence intensifies axonal degeneration remains untested.

Impaired LTMR signaling may also induce disability secondary to changes within the central nervous system (CNS) where neural network functions are susceptible to modification by altered activity of primary sensory neurons, including LTRMs (Chang et al., 2018; Housley et al., 2020b; Housley et al., 2021). Activity-dependent network modification is held responsible for wide ranging conditions (Wolpaw and Tennissen, 2001; Vucic et al., 2007; Van Zundert et al., 2008), including, for example, allodynia (Wolpaw and Tennissen, 2001) often reported in CIND (Cata et al., 2006; Tomita et al., 2019). These observations support the proposal that modification of CNS network modification further exacerbates and possibly prolongs disability originating from codependent intensification of LTMR firing abnormalities.

While the severity of CIND determines quality of life for people relying on chemotherapy to survive cancer, the conditions regulating severity remain obscure. Combining results from our preclinical studies demonstrates the capacity of chemo-cancer codependence to influence CIND severity. In an earlier report, we demonstrate that the magnitude of errors in a limb placement task relying on tactile and proprioceptive function covaries with the severity of LTMR encoding defects induced in rats by chemotherapy alone (Vincent et al., 2016). Specifically, a ∼50% static encoding error rate associates with a 10% movement error rate. In cOIN, the 2-fold magnification of the static encoding errors in combination with emergence of threshold and dynamic encoding errors is consistent with the doubling in movement error rate (Housley et al., 2020a). This observation suggests that managing the yet to be determined systemic processes altered by cancer has the potential to reduce disability without delaying, reducing, or suspending chemotherapy and its life saving benefits.

## Data Availability

The datasets presented in this study can be found in online repositories. The names of the repository/repositories and accession number(s) can be found below: https://github.com/nickh89/Mechanosensory-encoding-dysfunction-emerges-from-cancer-chemotherapy-interaction.
