# Supplementary material for: Mechanosensory encoding dysfunction emerges from cancer-chemotherapy interaction
Source: Front Mol Biosci. 2022 Nov 24;9:1017427. doi: 10.3389/fmolb.2022.1017427 (PMC9729348; doi:10.3389/fmolb.2022.1017427)
Supplement: Supplementary file 1 [file DataSheet1.PDF]

a.

Sensitivity/Threshold Functional Cluster		
1	Thr.L3	Length at which 1st action potential is detected on the 3rd slow ramp (mm)
2	Thr.L	Length at which 1st action potential is detected on the fast ramp (mm)
3	Thr.T1	Time at which 1st action potential is detected on the 1st slow ramp (ms)
4	Thr.F3	Muscle tendon force at which 1st action potential is detected on the 3rd slow ramp (dN)
5	Thr.F1	Muscle tendon force at which 1st action potential is detected on the 1st slow ramp (dN)
6	Thr.T	Time at which 1st action potential is detected on the fast ramp (ms)
7	Thr.F	Muscle tendon force at which 1st action potential is detected on the fast ramp (dN)
8	Thr.L1	Length at which 1st action potential is detected on the 1st slow ramp (mm)
9	Thr.T3	Time at which 1st action potential is detected on the 3rd slow ramp (ms)

Dyamic Signaling Functional Cluster		
10	Dyn.slp1	Slope of instantenous firing rate during the 1st slow ramp
11	Dyn.pfr1	Peak firing rate achieved during the 1st slow ramp (Hz, pps)
12	Dyn.spkNum	Number of action potentials (spikes) during the fast ramp (#)
13	Dyn.DI	Difference between the peak dynamic firing rate and the static firing rate during half hold (Hz)
14	Dyn.spkNum3	Number of action potentials (spikes) during the 3rd slow ramp (#)
15	Dyn.F	Force at which peak dynamic firing was achieved
16	Dyn.slp3	Slope of instantenous firing rate during the 3rd slow ramp
17	Dyn.spkNum1	Number of action potentials (spikes) during the 1st slow ramp (#)
18	Dyn.IB	Peak instantaneous high-frequency firing rate achieved immediately after stimulus (Hz)
19	Dyn.slp	Slope of instantenous firing rate during the fast ramp
20	Dyn.IFRdrop	Drop in instantaneous firing rate after the release on the slow ramps (Hz, pps)
21	Dyn.pfr3	Peak firing rate achieved during the 3rd slow ramp (Hz, pps)
22	Dyn.pfr	Peak firing rate achieved during the fast ramp (Hz, pps)

Static Signaling Functional Cluster		
23	Stat.mSfr	Instantenous firing rate midway through the static hold (Hz, pps)
24	Stat.afr	Average firing rate during the static hold (Hz, pps)
25	Stat.HzSTD	The standard deviation of instantaneous firing rate during the static hold
26	Stat.LSpkF	The muscle force at which the last action potential occurs during the static hold (dN).
27	Stat.slp	Slope of instantenous firing rate during the static hold.
28	Stat.LspkT	The time at which the last action potential occurs during the static hold (ms).
29	Stat.HzEnd	Instantenous firing rate during at the end of the static hold (Hz)
30	Stat.spkNum	Number of action potentials (spikes) during the static hold (#)

History Dependence Functional Cluster		
31	HxDep.RDR	Difference between the number of action potentials (spikes) in the 1st and 3rd slow ramp

b.

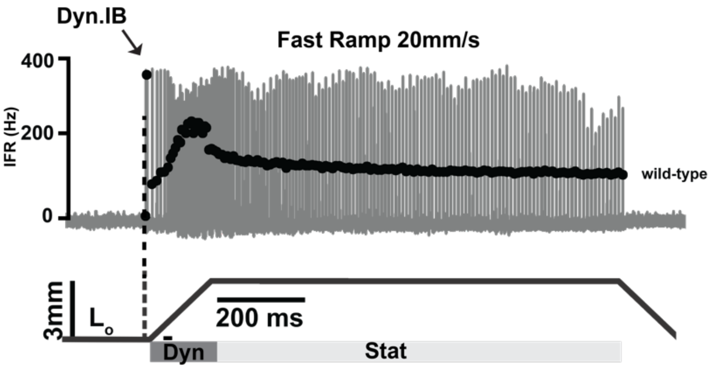

c.

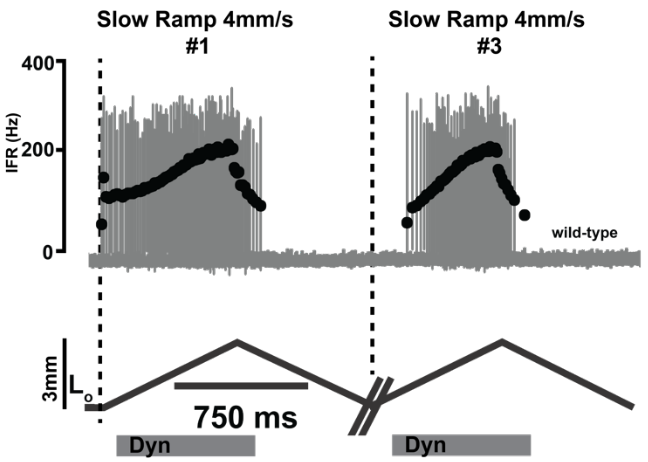

Figure S1. List of Parameters used for Neurophysiological Analyses. a, Measured and derived parameters were computed offline using custom written SPIKE2 scripts. Letters in the left column indicate the specific parameters included in analyses for each functional feature cluster. Thr: threshold (9 parameters); Dyn: dynamic (11 parameters); Stat: static (8 parameters); HxDep: history-dependent (1 parameter). **b**, Representative fast ramp-hold-release (3 mm at 20mm/s) trial from a proprioceptive neuron recorded from dorsal roots in *in vivo* electrophysiological experiments of a wild-type rat. **c**, Representative slow repeated ramp (3 mm at 4mm/s) trial from a proprioceptive neuron recorded from dorsal roots in *in vivo* electrophysiological experiments of a wild-type rat. Corresponding action potential trains and overlaid black circles indicate individual action potentials (spikes) and instantaneous firing rates (IFRs) of the responses. Dashed line marks the point of muscle stretch from background length (L_o) and indicates the starting point for threshold/sensitivity measurements. Boxes indicate dynamic (dark grey, 150 ms duration after stretch command onset) and static phases for analysis (light grey, 1 s duration after the dynamic phase).

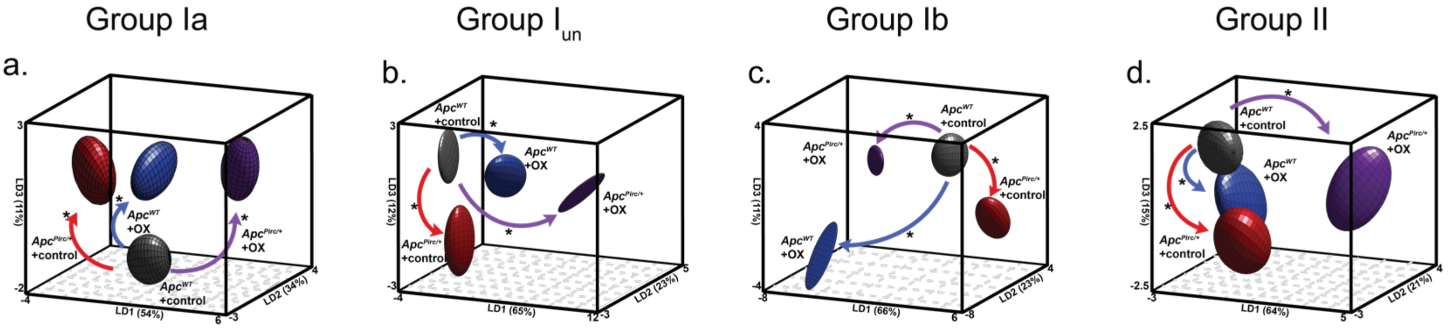

Supplemental Figure 2. Cancer-chemotherapy codependence exacerbates sensory dysfunction beyond that predicted by cancer or chemotherapy alone. Neuronal spiking parameters (n=31, averaged from four trials, from each neuron representing different features of sensory stimuli were subjected to linear discriminant (LD) analysis. Neuronal signaling was visualized in the new 3D composite space created by LD1-3 for each neuron class: spindle group Ia (**a**), unclassified spindle (**b**), group Ib (**c**), group II (**d**). 3D ellipsoids enclosing 68% of data were computed with least-squares elliptical fitting to emphasize differences between control and $Apc^{Pirc/+}$+OX neurons. Effects of independent (OX or $Apc^{Pirc/+}$) and combinatorial ($Apc^{Pirc/+}$+OX) treatment are indicated by curved arrows.

Supplemental Table 1. Summary statistics of each neuronal class and their respective signaling parameters.
